# Hepatic Arterial Infusion Chemotherapy plus either Toripalimab or Sorafenib as First‐line Therapy for Locally Advanced Hepatocellular Carcinoma: A Non‐comparative, Randomized Phase 2 Trial

**DOI:** 10.1002/mco2.70805

**Published:** 2026-06-08

**Authors:** Zhicheng Lai, Aojie Ge, Hanyue Ouyang, Zichao Wu, Yexing Huang, Qijiong Li, Li Xu, Binkui Li, Minshan Chen, Dongsheng Wen, Anna Kan, Ming Shi, Minke He

**Affiliations:** ^1^ Department of Liver Surgery Sun Yat‐sen University Cancer Center Guangzhou China; ^2^ State Key Laboratory of Oncology in South China; Guangdong Provincial Clinical Research Center for Cancer Sun Yat‐sen University Cancer Center Guangzhou China

**Keywords:** hepatic arterial infusion chemotherapy, hepatocellular carcinoma, locally advanced, sorafenib, toripalimab

## Abstract

This study was designed to provide prospective evidence for the combination of hepatic arterial infusion chemotherapy (HAIC) and toripalimab, which had suggested encouraging antitumor activity and safety in advanced hepatocellular carcinoma (HCC) previously. This single‐center, non‐comparative, randomized phase II study (NCT04135690) recruited locally advanced HCC participants (1:1) to receive HAIC plus either toripalimab (TorHAIC) or sorafenib (SoraHAIC) per 3 weeks. The primary endpoint was the progression‐free survival (PFS) rate at 6 months. Seventy‐two participants were randomly assigned to received TorHAIC (*n* = 36) or SoraHAIC (*n* = 36). The 6‐month PFS rate was 63.9% in the TorHAIC group and 61.1% in the SoraHAIC group. The median OS was 20.9 months in the TorHAIC group and 16.4 months in the SoraHAIC group, while the median PFS was 9.1 and 7.2 months, respectively. There were 12 participants (33.3%) developed grade 3–4 adverse events (AEs) in the TorHAIC group and 16 participants (44.4%) in the SoraHAIC group. Serious AEs were reported in two participants in the TorHAIC group and five participants in the SoraHAIC group. Our study suggested that the TorHAIC regimen had a favorable safety and efficacy profile in locally advanced HCC. However, these findings warrant validation in a phase III trial.

## Graphical abstract

This phase II study explored the efficacy and safety of HAIC plus toripalimab in locally advanced HCC. The primary endpoint was the PFS rate at 6 months. The 6‐month PFS rate was 63.9% in the TorHAIC group and 61.1% in the SoraHAIC group. Treatment with the TorHAIC regimen resulted in a median OS of 20.9 months and a median PFS of 9.1 months. TorHAIC regimen was associated with a low incidence of AEs.

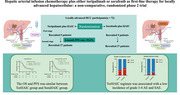

## 1 Introduction

Globally, hepatocellular carcinoma (HCC) is the leading cause of cancer‐related death, and approximately 54.9% of HCC patients are diagnosed at an advanced stage during their initial presentation in China [1, 2]. The first‐line treatment for advanced HCC is centered on combination systemic therapy, which integrates immune checkpoint inhibitors (ICIs) with anti‐angiogenic agents as represented by the atezolizumab plus bevacizumab [3, 4, 5]. Considering the leading cause of mortality in advanced HCC is progression of the intrahepatic tumor, effective control of intrahepatic lesions is critical for improving patients’ prognosis. Therefore, locoregional therapies have also played a crucial role in the management of advanced HCC [6].

As a locoregional therapy, hepatic arterial infusion chemotherapy (HAIC) delivers sustained, high‐concentration exposure of chemotherapeutic agents within tumor, thereby reducing tumor burden [7]. In published series of studies, HAIC have demonstrated promising antitumor activity and improved prognosis in the intermediate‐advanced‐stage HCC [7, 8, 9]. HAIC also induces tumor antigens release and promotes the infiltration of CD8^+^ T cells into tumor microenvironment [10]. Several studies, including the LEAP‐012 trial, have demonstrated that locoregional therapy can significantly enhance the antitumor activity of anti‐angiogenic agents and ICIs [11, 12, 13]. Therefore, the triple combination regimen of locoregional therapy, anti‐angiogenic agents and ICI has been widely promoted in China [14, 15].

The triple combination regimen has significantly prolonged survival in patients with advanced HCC. However, this benefit is accompanied by a significantly increased incidence of treatment‐related adverse events (AEs) [16]. Therefore, for locally advanced HCC, reducing the types of agents might help decrease the incidence of AEs while ensuring treatment efficacy.

Our previous randomized trial demonstrated that HAIC plus sorafenib significantly improved prognosis in patients with advanced HCC compared with sorafenib monotherapy, albeit with an increased incidence of AEs [8]. Notably, greater reductions in the risk of death or disease progression were observed in patients with low tumor burden such as those with a tumor diameter <10 cm or fewer than three tumors. Our team has also reported that HAIC combined with toripalimab (a programmed cell death protein‐1 [PD‐1] inhibitors) significantly improved survival in patients with advanced HCC compared with lenvatinib monotherapy, without increasing the incidence of grade 3–4 AEs [17]. However, there is currently no prospective study supporting the efficacy and safety of HAIC combined with toripalimab in locally advanced HCC.

Therefore, we initiated this prospective, non‐comparative, randomized phase 2 trial to evaluate the efficacy and safety of HAIC combined with toripalimab in locally advanced HCC.

## 2 Results

### 2.1 Participants

**FIGURE 1:**
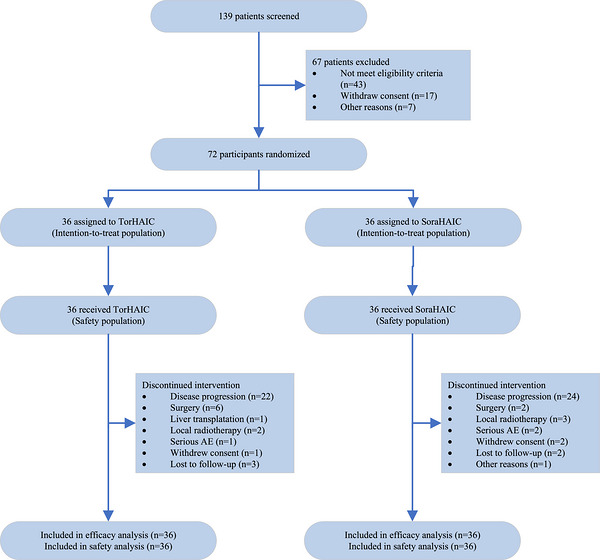
TorHAIC referred to HAIC plus toripalimab, while SoraHAIC referred to HAIC plus sorafenib.

From February 4, 2020, to December 17, 2021, a total of 139 patients from Sun Yat‐sen University Cancer Center were screened to determine their eligibility for the trial, and 72 participants were randomly assigned to receive TorHAIC (*n* = 36) or SoraHAIC (*n* = 36) (Figure 1).

As of the data cutoff date (December 31, 2024), the median follow‐up duration was 45.2 months (Interquartile range [IQR], 36.9–49.3 months). The mean age of the participants was 51 years. Sixty‐six participants (91.7%) were male, and 65 (90.3%) had HBV infection. The mean tumor diameter was 9.9 cm. Vp1‐2 and Vp3 portal vein tumor thrombosis was present in 46 (63.9%) and 26 (36.1%) participants, respectively. Detailed baseline characteristics of the two groups are presented in Table 1.

**TABLE 1:** Baseline characteristics.

	TorHAIC (*n* = 36)	SoraHAIC (*n* = 36)
Age		
≤50	16 (44.4%)	16 (44.4%)
>50	20 (55.6%)	20 (55.6%)
Sex		
Male	33 (91.7%)	33 (91.7%)
Female	3 (8.3%)	3 (8.3%)
ECOG score		
0	23 (63.9%)	20 (55.6%)
1	13 (36.1%)	16 (44.4%)
HBV infection		
Negative	4 (11.1%)	3 (8.3%)
Positive	32 (88.9%)	33 (91.7%)
ALT, median (IQR), U/L	42.5 (24.2–71.5)	51.2 (30.6–77.6)
AST, median (IQR), U/L	56.0 (35.3–96.1)	59.0 (44.3–97.3)
TBIL, median (IQR), µmol/L	13.8 (10.1–18.9)	16.6 (13.2–22.3)
ALB, median (IQR), g/dL	41.3 (36.2–43.6)	40.9 (38.5–43.3)
PT, median (IQR), s	12.5 (11.8–13.0)	12.1 (11.6–12.7)
AFP, ng/mL		
≤400	13 (36.1%)	12 (33.3%)
>400	23 (63.9%)	24 (66.7%)
Maximum tumor diameter (SD), cm	9.86 (3.82)	9.98 (3.06)
≤10	23 (63.9%)	22 (61.1%)
>10	13 (36.1%)	14 (38.9%)
Tumor number		
≤3	21 (58.3%)	22 (61.1%)
>3	15 (41.7%)	14 (38.9%)
6‐and‐12 score		
<6	1 (2.8%)	2 (5.6%)
6‐12	18 (50.0%)	17 (47.2%)
>12	17 (47.2%)	17 (47.2%)
PVTT		
Vp1‐2	25 (69.4%)	21 (58.3%)
Vp3	11 (30.6%)	15 (41.7%)
HVTT		
Yes	5 (13.9%)	9 (25.0%)
No	31 (86.1%)	27 (75.0%)

### 2.2 Efficacy

**FIGURE 2:**
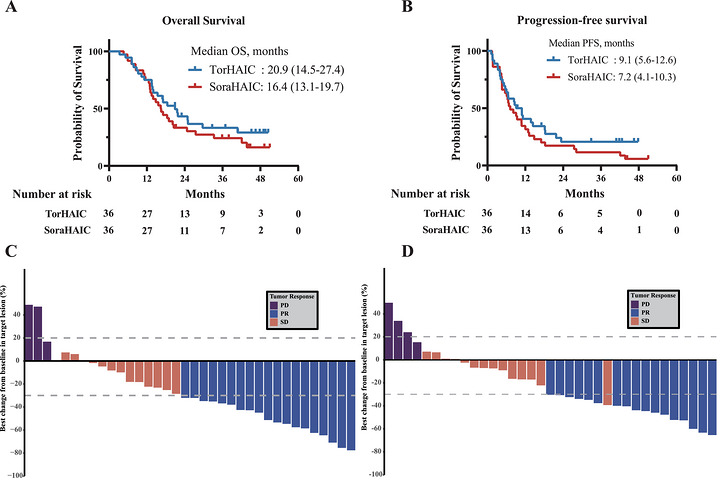
Survival analysis and waterfall plot of tumor response per RECIST v1.1. (A) Kaplan‒Meier curves of overall survival and (B) progression‐free survival of participants in TorHAIC and SoraHAIC groups. (C) Best change from baseline in target lesion in the TorHAIC group. One participant was assessed as PD due to the appearance of new intrahepatic lesions, despite a less than 20% increase in target lesions; one participant was found to have disease progression at a local hospital after one treatment cycle and did not return to our center for subsequent imaging follow‐up or treatment, and was therefore evaluated as PD despite the absence of clearly documented changes in target lesions. (D) Best change from baseline in target lesion in the SoraHAIC group. One participant was assessed as PD due to the appearance of new intrahepatic lesions, despite a less than 20% increase in target lesions; In one participant who had no distant metastasis at baseline, pulmonary metastases were identified while the hepatic target lesion achieved a partial response. Therefore, the best overall response was classified as stable disease.

In the TorHAIC group, 5 of the 9 participants enrolled in the first stage achieved PFS exceeding 6 months, thus meeting the criteria for proceeding to the second stage. Overall, the 6‐month PFS rate was 63.9% (95% CI, 47.1%–79.3%) in the TorHAIC group, meeting the primary endpoint, while it was 61.1% (95% CI, 46.2%–78.9%) in the SoraHAIC group. The median OS was 20.9 months (95% CI, 14.5–27.4) in the TorHAIC group and 16.4 months (95% CI, 13.1–19.7) in the SoraHAIC group (Figure 2A). The OS rates for the TorHAIC group at 12, 24, and 36 months were 75%, 36.1%, and 25%, respectively, while the corresponding rates for the SoraHAIC group were 75%, 30.6%, and 19.4%. The median PFS was 9.1 months (95% CI, 5.6–12.6) and 7.2 months (95% CI, 4.1–10.3) in the TorHAIC and SoraHAIC groups, respectively (Figure 2B). For participants with a tumor response of stable disease (SD) or progression disease (PD), the median OS was 13.8 months and the median PFS was 4.8 months in the TorHAIC group; the corresponding values were 12.9 months and 4.6 months in the SoraHAIC group (Figure S1A,S1B). Furthermore, according to the six‐and‐twelve score system for HCC [18], participants with low intrahepatic tumor burden demonstrated longer OS and PFS in both the TorHAIC and SoraHAIC groups (Figure S2A–S2F).

According to RECIST v1.1, the ORR was 52.8% (95% CI, 35.5%–69.6%; *n* = 19) in the TorHAIC group, whereas 47.2% (95% CI, 30.4%–64.5%; *n* = 17) in the SorHAIC group. Among responders, the median DoR was 9.8 months in the TorHAIC group and 6.8 months in the SorHAIC group (Figure S3A,S3B). The mean reduction in target lesion size from baseline was 50.9% (SD, 14.7%) in the TorHAIC group (Figure 2C) and 44.5% (SD, 11.2%) in the SorHAIC group (Figure 2D). Moreover, the ORR was 69.4% (95% CI, 51.9%–83.7%; *n* = 25) for the TorHAIC group and 52.8% (95% CI, 35.5%–69.6%; *n* = 19) for the SoraHAIC group, as assessed by mRECIST criteria. The corresponding CR rates were 19.4% and 11.1%, respectively. Detailed tumor response data are presented in Table 2. Our results suggested that, in both groups, participants who achieved PR might have the most favorable OS and PFS, followed by those with SD, while participants with PD might have the poorest outcomes (Figure S4).

**TABLE 2:** Tumor response.

	RECIST v1.1		mRECIST	
Overall	TorHAIC (*n* = 36)	SoraHAIC (*n* = 36)	TorHAIC (*n* = 36)	SoraHAIC (*n* = 36)
CR	0	0	7 (19.4%)	4 (11.1%)
PR	19 (52.8%)	17 (47.2%)	18 (50.0%)	15 (41.7%)
SD	13 (36.1%)	14 (38.9%)	8 (22.2%)	12 (33.3%)
PD	4 (11.1%)	4 (11.1%)	2 (5.6%)	4 (11.1%)
NA	0	1 (2.8%)	0	1 (2.8%)
ORR	19 (52.8%)	17 (47.2%)	25 (69.4%)	19 (52.8%)
DCR	32 (88.9%)	31 (86.1%)	33 (91.7%)	31 (86.1%)

### 2.3 Safety

All participants in this study experienced AEs of any grade, and there were no treatment‐related death in this study. In the TorHAIC group, 12 participants (33.3%) developed grade 3–4 AEs, while 16 participants (44.4%) in the SoraHAIC group experienced grade 3–4 AEs. Specifically, the grade 3–4 AEs in the TorHAIC group were elevated AST (16.7%), neutropenia (16.7%), elevated ALT (11.1%), and thrombocytopenia (2.8%). In the SoraHAIC group, the grade 3–4 AEs consisted of elevated AST (27.8%), neutropenia (13.9%), thrombocytopenia (11.1%), elevated ALT (8.3%), and hyperbilirubinemia (2.8%). Additionally, in the TorHAIC group, 3 participants developed grade 1–2 immune‐related dermatitis and 1 participant experienced an allergic reaction to toripalimab. Serious adverse events (SAEs) were reported in two participants in the TorHAIC group, including laryngeal edema due to oxaliplatin allergy (*n* = 1) and thrombocytopenia (*n* = 1). In the SoraHAIC group, five participants experienced SAEs, which were attributed to impaired myeloid function (*n* = 3), renal impairment (*n* = 1), and upper gastrointestinal bleeding (*n* = 1). Dose modifications were required in both groups. Five participants (13.9%) in the SoraHAIC group required sorafenib dose reduction due to toxicity, while seven participants (19.4%) in each group required FOLFOX reduction because of decreased tumor vascularity or chemotherapy toxicity. Treatment delayed due to AEs were also recorded: HAIC was interrupted in 3 (8.3%) and 5 participants (13.9%) in the TorHAIC and SoraHAIC groups, respectively, while toripalimab and sorafenib were interrupted in 6 (16.7%) and 10 (27.8%) participants, respectively (Table S1). A detailed summary of AEs is provided in Table 3.

**TABLE 3:** Treatment‐related adverse events.

	TorHAIC (*n* = 36)				SoraHAIC (*n* = 36)			
	Any grade	Grade 1–2	Grade 3	Grade 4	Any grade	Grade 1–2	Grade 3	Grade 4
Hypertension	3 (8.3%)	3 (8.3%)	0	0	9 (25.0%)	8 (22.2%)	1 (2.8%)	0
Fatigue	8 (21.2%)	8 (21.2%)	0	0	14 (38.9%)	14 (38.9%)	0	0
Fever	3 (8.3%)	3 (8.3%)	0	0	5 (13.9%)	5 (13.9%)	0	0
Sensory neuropathy	5 (13.9%)	5 (13.9%)	0	0	4 (11.1%)	4 (11.1%)	0	0
Abdominal pain	7 (19.4%)	7 (19.4%)	0	0	10 (27.8%)	10 (27.8%)	0	0
Nausea	9 (25.0%)	9 (25.0%)	0	0	14 (38.9%)	13 (36.1%)	1 (2.8%)	0
Vomit	5 (13.9%)	5 (13.9%)	0	0	12 (33.3%)	12 (33.3%)	0	0
Diarrhea	5 (13.9%)	5 (13.9%)	0	0	8 (22.2%)	7 (19.4%)	1 (2.8%)	0
Hand‐foot syndrome	0	0	0	0	8 (22.2%)	8 (22.2%)	0	0
Neutropenia	15 (41.7%)	9 (25.0%)	4 (11.1%)	2 (5.6%)	24 (66.7%)	19 (52.8%)	4 (11.1%)	1 (2.8%)
Anemia	14 (38.9%)	14 (38.9%)	0	0	12 (33.3%)	12 (33.3%)	0	0
Thrombocytopenia	14 (38.9%)	13 (36.1%)	1 (2.8%)	0	21 (58.3%)	17 (47.2%)	3 (8.3%)	1 (2.8%)
Elevated ALT	21 (58.3%)	17 (47.2%)	4 (11.1%)	0	25 (69.4%)	22 (61.1%)	3 (8.3%)	0
Elevated AST	33 (91.7%)	27 (75.0%)	6 (16.7%)	0	34 (94.4%)	24 (66.7%)	10 (27.8%)	0
Hyperbilirubinemia	12 (33.3%)	12 (33.3%)	0	0	13 (36.1%)	12 (33.3%)	1 (2.8%)	0
Hypoalbuminemia	35 (97.2%)	35 (97.2%)	0	0	35 (97.2%)	35 (97.2%)	0	0
Immune‐related dermatitis	3 (8.3%)	3 (8.3%)	0	0	0	0	0	0
Grade 3–4	12 (33.3%)				16 (44.4%)			
Serious AE	2 (5.6%)				5 (13.9%)			

### 2.4 Treatment Administration

**FIGURE 3:**
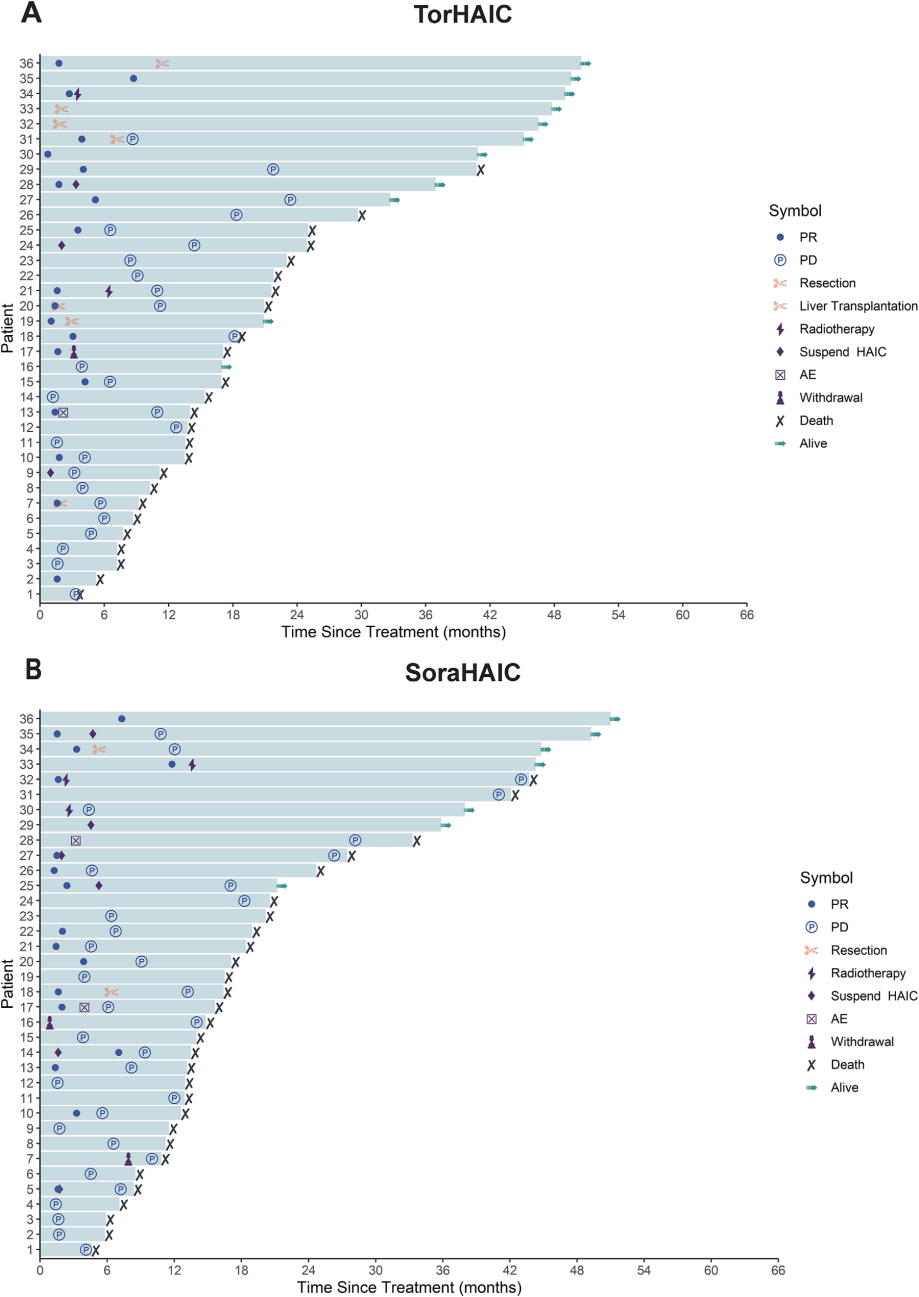
Swimmer plot of treatment duration. (A) Swimmer plot of TorHAIC group. (B) Swimmer plot of SoraHAIC group. The tumor response was assessed according to RECIST v1.1. The length of each bar represented the duration of follow‐up for each participant.

The swimmer plot of treatment duration and clinical outcome in this study is displayed in Figure 3A,B. The median number of HAIC sessions was 3 (IQR, 2–4) in both groups. In the TorHAIC group, the median number of toripalimab administrations was 7 (IQR, 4–10), while the median duration of sorafenib in the SoraHAIC group was 5 months (IQR, 3–9). There were 22 participants discontinued study treatment due to disease progression in the TorHAIC group, while 24 participants did so in the SoraHAIC group. Similarly, discontinuations due to SAEs were reported for one participant in the TorHAIC group (laryngeal edema due to oxaliplatin allergy) and two in the SoraHAIC group (renal impairment and upper gastrointestinal bleeding). Regarding subsequent therapies, surgery was performed in 7 participants (19.4%) in the TorHAIC group (including 1 who underwent liver transplantation) and in 2 participants (5.6%) in the SoraHAIC group. Anti‐angiogenic agents were administered to 23 participants (63.9%) in the TorHAIC group and 22 participants (61.1%) in the SoraHAIC group, whereas ICIs were administered in 14 (38.9%) and 16 (44.4%) participants, respectively. Detailed treatment administration for both groups was provided in the Table S2.

## 3 Discussion

To our knowledge, this is the first prospective trial assessing the combination of HAIC and ICIs in patients with advanced HCC. This study demonstrated that in locally advanced HCC, over 60% of participants receiving the TorHAIC regimen remained progression‐free at 6 months. Treatment with the TorHAIC regimen resulted in a median OS of 20.9 months and a median PFS of 9.1 months in this patient population, underpinned by high response rates, deep responses, and durable response duration. Moreover, TorHAIC regimen was associated with a low incidence of symptomatic AEs, indicating a favorable safety and tolerability profile.

In China, the combination of locoregional therapy with anti‐angiogenic agents and ICIs had promoted for advanced HCC due to its encouraging antitumor activity [12, 13]. However, the toxicities associated with multi‐drug regimens remains a concern, with high rates of grade 3–4 AEs reported [19]. Since HCC patients without high‐risk features or metastasis have a more favorable prognosis than those with such features [20], de‐escalation strategies by reducing drug categories in initial treatment merit further investigation. This study evaluated two de‐escalated regimens: one without anti‐angiogenic agents (TorHAIC) and one without ICIs (SoraHAIC), in locally advanced HCC patients. Given previous evidence supporting SoraHAIC in patients with portal vein tumor thrombus [8], the current analysis focuses primarily on the efficacy and safety of the TorHAIC regimen.

Previous retrospective studies had reported median OS ranging from approximately 18.0 to 40.4 months and median PFS between 10.0 and 22.1 months for regimens combining HAIC with ICIs [21, 22, 23]. Compared with our previous retrospective analysis, the TorHAIC regimen in this study demonstrated improved OS, which was likely associated with the lower tumor burden in the enrolled participants [17]. In this study, the median OS in both groups was less than 24 months, which was also shorter than that achieved with current standard first‐line therapies such as atezolizumab plus bevacizumab or nivolumab plus ipilimumab. This discrepancy was likely driven by the high proportion of participants with six‐and‐twelve score system exceeding 12 points. The Kaplan–Meier curves for both OS and PFS in this study revealed that the survival advantage of TorHAIC over SoraHAIC began to emerge after 12 months, possibly reflecting the long‐tail effect characteristic of ICIs. This observation was consistent with findings from the CheckMate 9DW trial [24], underscoring the long‐term survival benefit of ICIs in patients with advanced HCC. However, we also noted that among participants who did not achieve an objective response, the Kaplan–Meier curves for OS and PFS showed no separation between the two groups, and both median OS and PFS were unsatisfactory. Furthermore, we observed that among participants with six‐and‐twelve score system exceeding 12 points, the improvements in OS and PFS after treatment with the SoraHAIC or TorHAIC remained limited. On one hand, this suggested that treatment should be adjusted promptly if no significant response was observed initially in advanced HCC; on the other hand, it indicated that the antitumor activity of TorHAIC or SoraHAIC might be insufficient for locally advanced HCC with a high intrahepatic tumor burden, and that further triple combination regimen might be required.

The safety profile of the TorHAIC regimen in this study was consistent with previously reported data. Compared with the SoraHAIC group, the TorHAIC group suggested lower incidences of AEs accompanied by subjective symptoms, such as abdominal pain, hand‐foot syndrome, gastrointestinal reactions as well as lower rates of bone marrow toxicity. No cases of gastrointestinal bleeding were observed in the TorHAIC group, highlighting one of the safety advantages of avoiding anti‐angiogenic agents. However, immune‐related adverse events (irAEs) require close monitoring. Although immune‐related cutaneous toxicities are generally mild in severity, they may cause increased symptom burden and impair health‐related QoL [25]. On the other hand, cutaneous irAEs may also serve as a surrogate for clinical benefit [25]. In this study, two participants who developed immune‐related dermatitis achieved long‐term tumor response and survival. Therefore, accurate recognition of these skin changes might be important to ensure the continuation of ICIs therapy in such patients with favorable prognosis.

This study has several limitations. First, it was designed as a randomized, non‐comparative study, in which SoraHAIC was selected as the control group to check our hypotheses. Although SoraHAIC was not one of the first‐line treatment for advanced HCC, its efficacy and safety had been established in our previous phase III study. This choice allowed us to compare the effectiveness of toripalimab or sorafenib combined with HAIC while maintaining consistency in locoregional therapy. Since phase III study data were not yet available to validate triple combination regimen (HAIC, anti‐angiogenic agents and ICIs) in advanced HCC, this combination was not chosen for the control group of our study. In future studies, it is necessary to adopt the triple combination therapy as the control arm to explore the efficacy of drug de‐escalation strategy. Second, a subset of participants showed suboptimal response to either TorHAIC or SoraHAIC, indicating the need for further refinement in patient stratification when applying dual‐therapy regimens. Third, a key objective of this study was to investigate whether a dual‐therapy approach could improve quality of life without compromising efficacy in this patient population; however, the lack of patient‐reported QoL questionnaire data represents an important limitation. Fourth, the selection of the 6‐month PFS rate as the primary study endpoint presented an issue of insufficient follow‐up duration, rendering it less persuasive than overall survival for the assessment of therapeutic efficacy. We considered that patients with locally advanced HCC have a high risk of disease progression within 6 months, and choosing the 6‑month PFS rate as the endpoint allows for early differentiation of efficacy and directly measures the ability of study treatment to control disease progression. Finally, given the limited sample size and the absence of formal statistical comparisons, the efficacy and safety of the TorHAIC regimen in locally advanced HCC warrant further validation in phase III clinical trials.

In summary, this study suggested that the TorHAIC regimen might have a favorable safety and efficacy profile in patients with locally advanced HCC; however, this conclusion warrant validation in a subsequent phase III trial.

## 4 Methods

### 4.1 Study Design and Participants

This open‐label, non‐comparative, randomized phase II clinical trial was performed at one medical site in China. Protocol approval was obtained from the institutional review board or ethics committee (B2019‐110‐01). This study was conducted in accordance with the International Conference on Harmonization guidelines for Good Clinical Practice and the principles of the Declaration of Helsinki. All patients provided written informed consent. This trial was registered with ClinicalTrials.gov, NCT04135690.

Eligible participants for this study were required to be aged 18 years or older with the diagnosis of HCC was based on the American Association for the Study of Liver Diseases practice guidelines [26]. Participants had non‐high‐risk, locally advanced BCLC stage C HCC, defined as the absence of distant metastasis and not meeting the criteria for high‐risk features (Vp4, and/or bile duct invasion and/or tumor occupancy of 50% of the liver). Participants had not received previous treatment; had at least one measurable lesion per Response Evaluation Criteria in Solid Tumours version 1.1 (RECIST v1.1); a Child‐Pugh score of 5 or 6, an Eastern Cooperative Oncology Group performance status (ECOG‐PS) of 0 or 1 and adequate hematologic and organ function. Patients were excluded from this study if they had known fibrolamellar HCC, sarcomatoid HCC, or mixed cholangiocarcinoma and HCC; history of other invasive malignant diseases, hypersensitivity to relevant drugs, HIV, organ allograft and gastrointestinal bleeding within 30 days; previous treatment for HCC; evidence of hepatic decompensation including refractory ascites, acute variceal hemorrhage, hepatic encephalopathy, progressive jaundice, hepatorenal syndrome, coagulopathy and spontaneous bacterial peritonitis. Patients with active co‐infection with both hepatitis B virus (HBV) and hepatitis C virus (HCV) were also excluded. Full eligibility criteria are listed in the protocol.

### 4.2 Randomization

Participants were randomly assigned at a 1:1 ratio to receive either HAIC plus toripalimab (TorHAIC) or HAIC plus sorafenib (SoraHAIC). We used SoraHAIC as a control group to check our hypotheses, and as a calibration so that the populations in the two groups were similar. Randomization was performed centrally via a computer‐generated randomization sequence.

### 4.3 Procedures

The detailed procedures of HAIC were provided in the study protocol. The microcatheter was advanced into the hepatic artery on Day 1 in every cycle of treatment [8]. The participants were transferred to the inpatient ward for drug infusion via the hepatic artery as follows: oxaliplatin, 85 mg/m^2^ from hour 0 to 2 on day 1; leucovorin, 400 mg/m^2^ from hour 2 to 3 on Day 1; and fluorouracil, 400 mg/m^2^ bolus at hour 3 and 2400 mg/m^2^ over 24 h. HAIC was repeated every 3 weeks for up to six cycles. Toripalimab was administered 240 mg by intravenous infusion prior to HAIC every 3 weeks. Sorafenib was administered 400 mg orally twice daily. First treatment administration was given within 7 days after randomization. Participants with HBV infection in this study concomitantly received effective antiviral therapy. The dose reduction of sorafenib was allowed for the management of suspected treatment‐related adverse events. The dose of toripalimab was not subject to escalations or reductions. Dose delays were permitted for toripalimab due to adverse events until retreatment criteria were met. When imaging demonstrated an avascular tumor, HAIC administration would be suspended while the participants remained on‐study, continuing with either toripalimab monotherapy or sorafenib monotherapy.

Tumor assessments were done by contrast‐enhanced CT of the chest and CT or MRI of the abdomen. Baseline tumor assessments were performed within 2 weeks before randomization. And the laboratory tests were completed within 1 week before treatment initiation. After treatment initiation, participants were evaluated for tumor response every 6 weeks and laboratory tests were performed every 3 weeks until disease progression, treatment discontinuation, or initiation of subsequent therapy. The tumor response to guide ongoing study treatment decisions were assessed by investigators according to RECIST v1.1.

The subsequent therapy, criteria for dose reduction, and discontinuation of therapy were shown in Protocol.

### 4.4 Outcomes

The primary endpoint was the progression‐free survival (PFS) rate at 6 months, which was defined as the proportion of participants alive, assessable, and free from progression according to RECIST v1.1 at 6 months. The secondary endpoints were overall survival (OS), PFS, objective response rate (ORR), duration of response (DoR) and adverse events (AE). OS was defined as the time from randomization to death from any cause. PFS was defined as the time from randomization to disease progression according to RECIST v1.1 or death from any cause, which occurred first. ORR was defined as the proportion of participants whose best overall response was either a confirmed complete response (CR) or partial response (PR) according to RECIST v1.1. Best overall response was defined as the best response designation recorded between the date of randomization and first objectively documented progression or subsequent therapy, whichever occurred first. DoR was defined as the time between the date of first confirmed documented response of CR or PR and the date of the first documented tumor progression or death due to any cause, whichever occurred first. Adverse events were assessed according to the National Cancer Institute Common Terminology Criteria for Adverse Events (version 5.0).

### 4.5 Statistical Analysis

Sample size determination was based on the 6‐month PFS rate in patients with advanced HCC. The sample size was calculated using Simon's two‐stage design, with a one‐sided significance level (α) of 0.05 and a power of 0.8. The REFLECT trial demonstrated a 6‐month PFS rate of 30% in patients with advanced HCC treated with sorafenib monotherapy [27], whereas our previous trial had reported a 6‐month PFS rate of 55% for HAIC combined with sorafenib [8]. We anticipatedthat the 6‐month PFS rate for advanced HCC patients receiving HAIC combined with toripalimab would be not inferior to 55%. Therefore, in the first stage of this study, 9 participants would be enrolled per group. The study would proceed to the second stage only if PFS exceeds 6 months in 3 participants from the TorHAIC group. The second stage would enroll 26 additional participants, resulting in a theoretical total of 35 subjects per group across both stages. Accounting for an estimated 5% dropout rate, 36 subjects will actually be enrolled per group. The treatment regimen will be considered effective if 14 participants in the TorHAIC group achieve PFS exceeding 6 months.

Efficacy and safety were assessed in all participants who had been randomly assigned to treatment (the intention‐to‐treat population). No formal statistical comparison was planned between the two groups. The results are reported as the means (standard deviations [SDs]), numbers (%), or medians (95% confidence intervals [CIs]). We measured the estimated OS, PFS and DoR using the Kaplan–Meier method, described with median at specific timepoints with 95% CI. We used SPSS (version 25.0, RRID: SCR_002865) and R studio (R version 4.3.0) for all analyses.

## Author Contributions

Minke He had full access to all of the data in the study and takes responsibility for the integrity of the data and the accuracy of the data analysis. Zhicheng Lai, Aojie Ge, Hanyue Ouyang and Zichao Wu contributed equally to this study. Conceptualization: Minke He and Ming Shi. Data curation and formal analysis: Zhicheng Lai, Aojie Ge, Hanyue Ouyang, and Zichao Wu. Software and visualization: Zhicheng Lai, Aojie Ge, Hanyue Ouyang, and Yexing Huang. Validation: Zhicheng Lai, Minke He, Anna Kan, and Dongsheng Wen. Writing – original draft: Zhicheng Lai, Aojie Ge, and Zichao Wu. Writing – review & editing: Minke He Ming Shi, Anna Kan, and Dongsheng Wen. Investigation: Zhicheng Lai, Qijiong Li, and Li Xu. Project administration: Minshan Chen and Binkui Li. Supervision: Minke He and Ming Shi. All authors have read and approved the final manuscript.

## Funding Information

This study was funded by the National Key Research and Development Program of China (Grant No.: 2023YFA0915700), the National Natural Science Foundation of China (Grant Nos.: 82272890 and 82403938), the Postdoctoral Fellowship Program of CPSF (Grant No.: GZC20250977), the Cancer Innovation Research Program of Sun Yat‐sen University Cancer Center (Grant No.: CIRP‐SYSUCC‐0013), the Young Talents Program of Sun Yat‐sen University Cancer Center (Grant Nos.: YTP‐SYSUCC‐0071 and and YTP‐SYSUCC‐0077), and the Beijing iGandan Foundation (No. GDXZ‐08‐23).

## Ethics Statement

This trial was registered with ClinicalTrials.gov, NCT04135690. Protocol approval was obtained from the institutional review board or ethics committee of Sun Yat‐sen University Cancer Center (B2019‐110‐01). Written informed consent was obtained from all participants.

## Conflicts of Interest

The authors declare no conflicts of interest.

## Supporting information




**Supporting Figure S1**: Survival analysis in participants achieved progression/stable disease. (A) Kaplan‒Meier curves of overall survival and (B) progression‐free survival of participants achieved progression/stable disease in TorHAIC and SoraHAIC groups.
**Supporting Figure S2**: Survival analysis stratified by 6‐and‐12 score. (A) Kaplan‒Meier curves of overall survival and (B) progression‐free survival of all participants stratified by 6‐and‐12 score. (C) Kaplan‒Meier curves of overall survival and (D) progression‐free survival of TorHAIC group stratified by 6‐and‐12 score. (E) Kaplan‒Meier curves of overall survival and (F) progression‐free survival of SoraHAIC group stratified by 6‐and‐12 score.
**Supporting Figure S3**: Duration of response. (A) Kaplan‒Meier curves of duration of response according to RECIST v1.1 and mRECIST (B) in TorHAIC and SoraHAIC groups.
**Supporting Figure S4**: Survival analysis stratified by tumor response. (A) Kaplan‒Meier curves of overall survival and (B) progression‐free survival of TorHAIC group stratified by tumor response. (C) Kaplan‒Meier curves of overall survival and (D) progression‐free survival of SoraHAIC group stratified by tumor response.
**Supporting Table S1**: Dose modification and treatment delay.
**Table S2**: Treatment administration.

## Data Availability

The data that support the findings of this study are available on request from the corresponding author.
