# Supplementary material for: Hepatic Arterial Infusion Chemotherapy plus either Toripalimab or Sorafenib as First‐line Therapy for Locally Advanced Hepatocellular Carcinoma: A Non‐comparative, Randomized Phase 2 Trial
Source: MedComm (2020). 2026 Jun 8;7(6):e70805. doi: 10.1002/mco2.70805 (PMC13244076; doi:10.1002/mco2.70805)
Supplement: Supplementary file 1 — Supporting Figure S1: Survival analysis in participants achieved progression/stable disease. (A) Kaplan‒Meier curves of overall survival and (B) progression‐free survival of participants achieved progression/stable disease in TorHAIC and SoraHAIC groups. Supporting Figure S2: Survival analysis stratified by 6‐and‐12 score. (A) Kaplan‒Meier curves of overall survival and (B) progression‐free survival of all participants stratified by 6‐and‐12 score. (C) Kaplan‒Meier curves of overall survival and (D) progression‐free survival of TorHAIC group stratified by 6‐and‐12 score. (E) Kaplan‒Meier curves of overall survival and (F) progression‐free survival of SoraHAIC group stratified by 6‐and‐12 score. Supporting Figure S3: Duration of response. (A) Kaplan‒Meier curves of duration of response according to RECIST v1.1 and mRECIST (B) in TorHAIC and SoraHAIC groups. Supporting Figure S4: Survival analysis stratified by tumor response. (A) Kaplan‒Meier curves of overall survival and (B) progression‐free survival of TorHAIC group stratified by tumor response. (C) Kaplan‒Meier curves of overall survival and (D) progression‐free survival of SoraHAIC group stratified by tumor response. Supporting Table S1: Dose modification and treatment delay. Table S2: Treatment administration. [file MCO2-7-e70805-s001.pdf]

Original article

Hepatic arterial infusion chemotherapy plus either toripalimab or sorafenib as first-line therapy for locally advanced hepatocellular carcinoma: a non-comparative, randomized phase 2 trial

Running title

TorHAIC vs SoraHAIC in advanced HCC.

Zhicheng Lai[1,2#]. laizc@sysucc.org.cn;

Aojie Ge[1,2#]. geaojie@sysucc.org.cn;

Hanyue Ouyang[1,2#]. ouyhy@sysucc.org.cn;

Zichao Wu[1,2#]. wuzc1@sysucc.org.cn;

Yexing Huang[1,2]. huangyx3@sysucc.org.cn;

Qijiong Li[1,2]. liqj@sysucc.org.cn;

Li Xu[1,2]. xuli@sysucc.org.cn;

Binkui Li[1,2]. libk@sysucc.org.cn;

Minshan Chen[1,2]. chenmsh@sysucc.org.cn;

Dongsheng Wen[1,2*]. wends@sysucc.org.cn;

Anna Kan[1,2*]. annakan@sysucc.org.cn;

Ming Shi[1,2*]. shiming@sysu.edu.cn;

Minke He[1,2*]. hemk@sysucc.org.cn;

Affiliations

[1]Department of Liver Surgery, Sun Yat-sen University Cancer Center; Guangzhou, China.

[2]State Key Laboratory of Oncology in South China; Guangdong Provincial Clinical Research Center for Cancer, Sun Yat-sen University Cancer Center; Guangzhou, China.

[#]Equal contribution.

[*]To whom correspondence should be addressed.

Address for Correspondence

Prof. Minke He, Department of Liver Surgery, Sun Yat-sen University Cancer Center, Guangzhou, 510060, P. R. China. Telephone: 020-87343938; Fax: 020-87343585; Email: hemk@sysucc.org.cn.

Prof. Ming Shi, Department of Liver Surgery, Sun Yat-sen University Cancer Center, Guangzhou, 510060, P. R. China. Telephone: 020-87343938; Fax: 020-87343585; Email: shiming@sysu.edu.cn.

Prof. Anna Kan, Department of Liver Surgery, Sun Yat-sen University Cancer Center, Guangzhou, 510060, P. R. China. Telephone: 020-87343938; Fax: 020-87343585; Email: annakan@sysucc.org.cn.

Prof. Dongsheng Wen, Department of Liver Surgery, Sun Yat-sen University Cancer Center, Guangzhou, 510060, P. R. China. Telephone: 020-87343938; Fax: 020-87343585; Email: wends@sysucc.org.cn.

Highlights

Given the established use of HAIC combined with immune checkpoint inhibitors (ICIs) and anti-angiogenic agents in advanced HCC, prospective data are lacking regarding the efficacy and safety of HAIC plus ICIs as a treatment de-escalation strategy for locally advanced HCC. Our study suggested that treatment with the HAIC plus toripalimab resulted in improved OS and PFS in this patient population, underpinned by high response rates, deep responses, and durable response duration. Moreover, TorHAIC regimen was associated with a low incidence of symptomatic adverse events, indicating a favorable safety and tolerability profile.

Legends for supplementary figures

Figure S1. Survival analysis in participants achieved progression/stable disease.

(A) Kaplan–Meier curves of overall survival and (B) progression-free survival of participants achieved progression/stable disease in TorHAIC and SoraHAIC groups.

Figure S2. Survival analysis stratified by 6-and-12 score.

(A) Kaplan–Meier curves of overall survival and (B) progression-free survival of all participants stratified by 6-and-12 score. (C) Kaplan–Meier curves of overall survival and (D) progression-free survival of TorHAIC group stratified by 6-and-12 score. (E) Kaplan–Meier curves of overall survival and (F) progression-free survival of SoraHAIC group stratified by 6-and-12 score.

Figure S3. Duration of response.

(A) Kaplan–Meier curves of duration of response according to RECIST v1.1 and mRECIST (B) in TorHAIC and SoraHAIC groups.

Figure S4. Survival analysis stratified by tumor response.

(A) Kaplan–Meier curves of overall survival and (B) progression-free survival of TorHAIC group stratified by tumor response. (C) Kaplan–Meier curves of overall survival and (D) progression-free survival of SoraHAIC group stratified by tumor response.

Table S1. Dose modification and treatment delay.

	TorHAIC group (n=36)	SoraHAIC (n=36)
**Dose reduction**		
Sorafenib	-	5 (13.9%)
Oxaliplatin and fluorouracil	7 (19.4%)	7 (19.4%)
**Treatment delayed due to AEs**		
HAIC	3 (8.3%)	5 (13.9%)
Toripalimab	6 (16.7%)	-
Sorafenib	-	10 (27.8%)

Table S2. Treatment administration

	TorHAIC group (n=36)	SoraHAIC group (n=36)
**Study treatment, median (IQR)**		
HAIC cycle	3 (2-4)	3 (2-4)
PD-1 cycle	7 (4-10)	-
Duration of sofafenib, months	-	5 (3-9)
**Number of participants who received subsequent treatments**		
HAIC	3 (8.3%)	3 (8.3%)
TACE	15 (41.7%)	18 (50.0%)
Liver transplantation	1 (2.8%)	0
Resection	6 (16.7%)	2 (5.6%)
Radiotherapy	5 (13.9%)	6 (16.7%)
Chemotherapy	1 (2.8%)	0
Anti-angiogenic agents	23 (63.9%)	22 (61.1%)
Immune checkpoint inhibitors	14 (38.9%)	16 (44.4%)

Figure S1

A

Overall Survival in Participants wtih Progression/Stable Disease

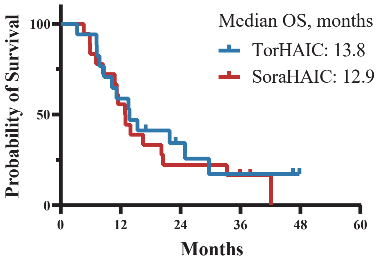

B

Progression-free Survival in Participants with Progression/Stable Disease

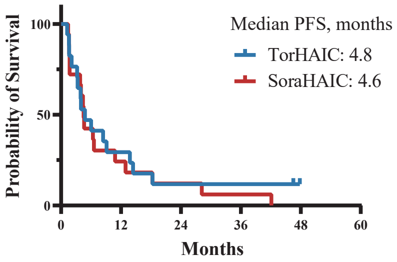

Figure S2

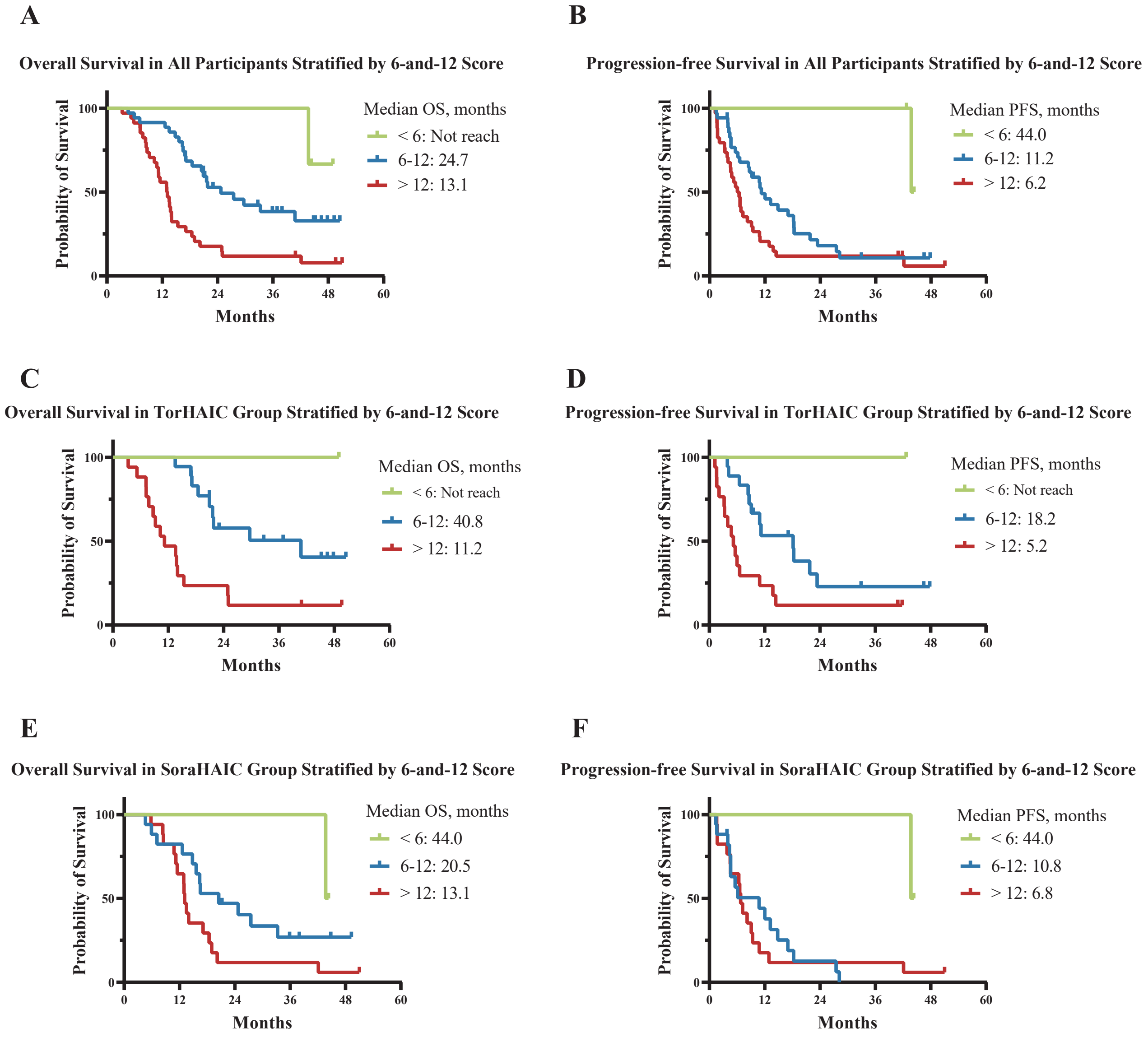
A
Overall Survival in All Participants Stratified by 6-and-12 Score
Probability of Survival
100
50
0
Months
0 12 24 36 48 60
Median OS, months
< 6: Not reach
6-12: 24.7
> 12: 13.1
B
Progression-free Survival in All Participants Stratified by 6-and-12 Score
Probability of Survival
100
50
0
Months
0 12 24 36 48 60
Median PFS, months
< 6: 44.0
6-12: 11.2
> 12: 6.2
C
Overall Survival in TorHAIC Group Stratified by 6-and-12 Score
Probability of Survival
100
50
0
Months
0 12 24 36 48 60
Median OS, months
< 6: Not reach
6-12: 40.8
> 12: 11.2
D
Progression-free Survival in TorHAIC Group Stratified by 6-and-12 Score
Probability of Survival
100
50
0
Months
0 12 24 36 48 60
Median PFS, months
< 6: Not reach
6-12: 18.2
> 12: 5.2
E
Overall Survival in SoraHAIC Group Stratified by 6-and-12 Score
Probability of Survival
100
50
0
Months
0 12 24 36 48 60
Median OS, months
< 6: 44.0
6-12: 20.5
> 12: 13.1
F
Progression-free Survival in SoraHAIC Group Stratified by 6-and-12 Score
Probability of Survival
100
50
0
Months
0 12 24 36 48 60
Median PFS, months
< 6: 44.0
6-12: 10.8
> 12: 6.8

Figure S3

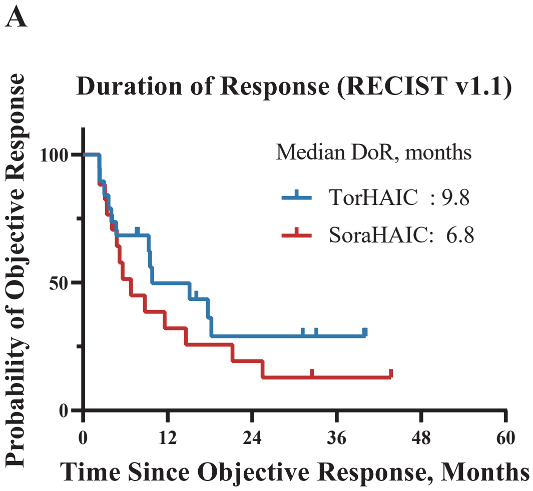
A
Duration of Response (RECIST v1.1)
Probability of Objective Response
100
50
0
Median DoR, months
TorHAIC : 9.8
SoraHAIC: 6.8
0
12
24
36
48
60
Time Since Objective Response, Months
B
Duration of Response (mRECIST)
Probability of Objective Response
100
50
0
Median DoR, months
TorHAIC : 10.6
SoraHAIC: 7.7
0
12
24
36
48
60
Time Since Objective Response, Months

Figure S4

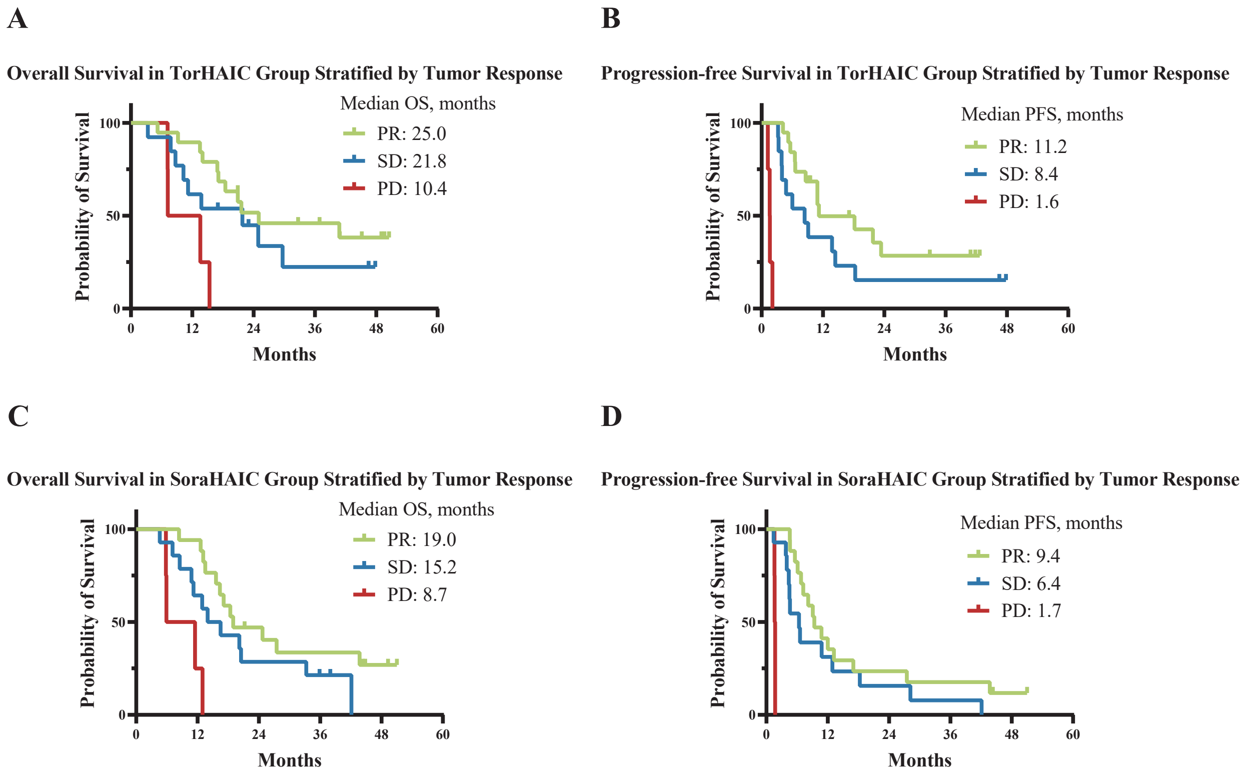

A
Overall Survival in TorHAIC Group Stratified by Tumor Response
Median OS, months
PR: 25.0
SD: 21.8
PD: 10.4
Probability of Survival
Months
B
Progression-free Survival in TorHAIC Group Stratified by Tumor Response
Median PFS, months
PR: 11.2
SD: 8.4
PD: 1.6
Probability of Survival
Months
C
Overall Survival in SoraHAIC Group Stratified by Tumor Response
Median OS, months
PR: 19.0
SD: 15.2
PD: 8.7
Probability of Survival
Months
D
Progression-free Survival in SoraHAIC Group Stratified by Tumor Response
Median PFS, months
PR: 9.4
SD: 6.4
PD: 1.7
Probability of Survival
Months

Study Protocol

Title: Hepatic Arterial Infusion Chemotherapy plus Toripalimab versus Hepatic Arterial Infusion Chemotherapy plus Sorafenib in the Treatment of Advanced Hepatocellular Carcinoma: A Prospective, Randomized, Non-comparative Study

Test Drug: Oxaliplatin, leucovorin, 5-fluorouracil; Sorafenib; Toripalimab

Principal Investigator: Ming Shi, MD, Professor

Study hospital： Sun Yat-sen University Cancer Center

Table of Contents

1. Introduction 4
1.1. Hepatic Arterial Infusion Chemotherapy plus Sorafenib for Advanced HCC 4
1.2 PD-1 Monoclonal Antibody in Solid Tumors 5
1.3 Hepatic Arterial Infusion Chemotherapy plus toripalimab for Advanced HCC 6
2. Objectives 6
2.1 Primary Objective 6
2.2 Secondary Objectives 7
3. Study Design and Sample Size 7
3.1 Study Design 7
3.2 Sample Size 7
4. Patients Population 8
4.1 Inclusion Criteria 8
4.2 Exclusion Criteria 9
5. Treatment and Administration 10
6. Dose Modification 12
6.1 Treatment Interruptions for toripalimab 12
6.2 Treatment Interruptions for HAIC 12
7. Study Procedures 13
7.1 Before treatment 13
7.2 Within Treatment 13
7.3 Follow-up 14
8. Efficacy 14
8.1 Evaluations 14
8.2 Endpoints 14
9. Safety Evaluations 15
9.1 Adverse Event 15
9.2 Serious Adverse Event 15
9.3 Attribution Definitions 16
9.4. Intensity of AE 16
10. Data Management 17
10.1 Data Collection 17
10.2 Statistical Analysis 17
10.3 Tumor Response 18
11. Ethical Considerations 18
11.1 Protection of Patients' Rights 18
11.2 Informed Consent 18
11.3 Protection of Personal Information and Identification of Patients 19
11.4 Conflicts of Interest 19

11.5 Institutional Review Board (IRB) 19
12. Appendices 19
12.1 Tumor Assessment 19
12.2 Definitions of Eastern Cooperative Oncology Group Performance Status 21
12.3 Child–Pugh Score* 22
12.4 BCLC Staging System 23
13. Reference 24

1. Introduction

Hepatocellular carcinoma (HCC) accounts for 55% of global cases in China, ranking as the second leading cause of cancer-related mortality nationwide[1]. HCC is characterized by insidious onset and rapid progression. Approximately 50% of HCC patients are initially diagnosed with advanced-stage disease[2]. The prognosis for these patients is extremely poor, with a median natural overall survival ranging from only 4.2 to 7.9 months[3]. For these patients, sorafenib is recommended as the standard treatment[5]; however, sorafenib therapy merely extends median overall survival to 6.5–10.7 months[3], which remains unsatisfactory. Sorafenib is particularly less effective in patients with hepatitis B-related HCC or those from the Asia-Pacific region[8].

1.1. Hepatic Arterial Infusion Chemotherapy plus Sorafenib for Advanced HCC

In Asia, transarterial chemoembolization (TACE) is widely used for treating advanced HCC[1][9], with numerous studies reporting its safety and efficacy[10]. Unlike TACE, hepatic arterial infusion chemotherapy (HAIC) is based on the continuous, prolonged intra-arterial infusion of high-dose chemotherapeutic agents without employing embolic agents. Multiple phase II clinical trials have demonstrated the superiority of HAIC over TACE in advanced HCC[15]. Consequently, HAIC is strongly recommended for advanced HCC in the consensus guidelines of the Chinese, Japanese, and Korean liver associations[7]. Compared to TACE, HAIC offers several advantages: 1) Unlike the single administration in TACE, HAIC can be sustained over several days, significantly increasing the total chemotherapeutic dose delivered and meeting the dose requirements for high tumor burden; 2) Continuous arterial infusion markedly prolongs the duration of action of high-concentration chemotherapeutic drugs; 3) The avoidance of embolic agents precludes adverse reactions such as post-embolization syndrome and ectopic embolism, enhancing safety and efficacy[17]; 4) HAIC can be applied to tumors throughout the entire liver.

The EACH study demonstrated the efficacy and safety of systemic chemotherapy using the FOLFOX regimen (oxaliplatin, leucovorin, and fluorouracil) in advanced HCC[19]. Preliminary research has also confirmed the efficacy of hepatic arterial infusion with FOLFOX (FOLFOX-HAIC) in advanced HCC[20]. Although HAIC significantly reduces tumor burden, patients often cannot continue HAIC after multiple sessions due to challenges like catheterization difficulties and chemotherapeutic adverse effects. Therefore, selecting appropriate combination therapies as ongoing treatment options holds promise for further prolonging patient survival. Our previous studies also confirmed that HAIC combined with sorafenib is a safe and effective treatment modality, yet the median progression-free survival was only 6.7 months[20].

1.2 PD-1 Monoclonal Antibody in Solid Tumors

In recent years, immunotherapeutic agents—anti-programmed death-1 (PD-1) receptor antibodies have achieved remarkable efficacy in various advanced cancers by overcoming immune suppression within patients to reactivate immune cells for tumor cell killing[22]. This represents a novel anti-cancer treatment modality beyond surgery, chemotherapy, and radiotherapy. Furthermore, it has been approved by the US Food and Drug Administration (FDA) for treating multiple advanced cancers, such as melanoma and renal cell carcinoma[23]. China's National Medical Products Administration (NMPA) facilitated the rapid approval of Nivolumab injection via a priority review green channel, marking the formal entry of cancer treatment in China into the era of tumor immunotherapy.

However, the efficacy and safety profiles of PD-1 monoclonal antibodies vary among different manufacturers, and their effectiveness is not consistently replicable within the same tumor type[27]. Toripalimab, developed by Shanghai Junshi Biosciences Co., Ltd., is the first domestically produced PD-1 monoclonal antibody approved for marketing by the NMPA. Compared to Nivolumab and Pembrolizumab, toripalimab demonstrates stronger stimulation of $CD4^+$ and $CD8^+$ T-cell proliferation, similar pharmacokinetics, lower immunogenicity, a different epitope, higher affinity, comparable safety, and

superior in vivo efficacy. Moreover, the price of toripalimab is less than one-third that of imported products, making it more accessible for patients in China. However, for immunotolerant malignancies like HCC, the clinical efficacy of PD-1 monoclonal antibodies remains limited[29]. Additionally, a recent phase 3 study of pembrolizumab failed to meet its primary endpoints (data not yet published). Therefore, there is a necessity to explore new treatment strategies for advanced HCC.

1.3 Hepatic Arterial Infusion Chemotherapy plus toripalimab for Advanced HCC

Combinations of chemotherapy and PD-1 monoclonal antibodies have demonstrated synergistic effects in both basic and clinical research[31]. This combination therapy can induce immunogenic cell death, activate macrophages, and upregulate recognition molecules, thereby enhancing the function of antigen-presenting cells within the tumor microenvironment[32]. Furthermore, studies have reported that chemotherapy can induce PD-L1 expression on tumor cells, modulating their immune function[34]. Consequently, the combination of HAIC and toripalimab is anticipated to achieve superior therapeutic outcomes.

However, no prospective studies had confirmed the efficacy and safety of hepatic arterial infusion chemotherapy (HAIC) combined with toripalimab in the treatment of advanced HCC. Therefore, we plan to conduct a prospective, randomized, non-comparative phase II study to investigate the efficacy and safety of HAIC combined with toripalimab in the treatment of advanced hepatocellular carcinoma.

2. Objectives

2.1 Primary Objective

Progression-free survival rate at 6 months

Progression-free survival rate at 6 months was defined as the proportion of patients alive, assessable, and free from progression at 6 months by independent radiologic review according to RECIST v1.1 or death from any cause.

2.2 Secondary Objectives

Overall survival, defined as the time from treatment initiation to death from any cause.
Progression-free survival, defined as the time from treatment initiation to disease progression according to RECIST v1.1 or death from any cause.
Objective response rate, the proportion of patients with complete response or partial response according to RECIST v1.1.
Duration of response, defined as the time between the date of first confirmed documented response of CR or PR and the date of the first documented tumour progression or death due to any cause, whichever occurred first.
Adverse events were assessed according to the National Cancer Institute Common Terminology Criteria for Adverse Events version 5.0.

3. Study Design and Sample Size

3.1 Study Design

This is a randomized, non-comparative phase II-trial conducted at the Sun Yat-sen university Cancer Center. Patients will be randomly assigned in a 1:1 ratio to either the experimental group (HAIC plus toripalimab) or the control group (HAIC plus sorafenib). The study will enroll patients in two stages. Provided that the 6-month PFS rate in the experimental group meets the predefined criteria in the first stage, the study will proceed to the second stage; otherwise, the trial will be discontinued.

3.2 Sample Size

This study is a randomized controlled, non-comparative phase II clinical trial designed to evaluate the 6-month PFS rate of HAIC combined with toripalimab in patients with advanced HCC. A significance level of $\alpha = 0.05$ and a statistical power of 80% were adopted. The REFLECT trial reported a 6-month PFS rate of 30% in advanced HCC patients treated with sorafenib[3]. Previous research by our team

indicated a 6-month PFS rate of 55% for HAIC combined with sorafenib in this patient population[36]. We anticipate that the 6-month PFS rate for HAIC combined with toripalimab will be no less than 55%. In the first stage, 9 patients per group were enrolled. At least 3 patients in the experimental group achieving PFS beyond 6 months were required to proceed to the second stage. An additional 26 patients were recruited in the second stage, resulting in a theoretical total of 35 patients per group across both stages. Accounting for a 5% dropout rate, 36 patients per group were actually enrolled. The treatment regimen would be considered effective if at least 14 patients in the experimental group achieved PFS beyond 6 months.

4. Patients Population

Three populations were used to analyze the data: the intention-to-treat population, the per-protocol population, and the safety population. The intention-to-treat population was defined as all enrolled patients and the per-protocol population was defined as all patients who had received at least one dose of study medication and had at least one available post-baseline tumor assessment. The safety population included the enrolled patients who received at least one dose of study medication but excluded the patients without any safety data. We analyzed efficacy in the intention-to-treat population and the per-protocol population. Patients without any post-baseline assessment would be defined as not assessable for efficacy and ruled out from the per-protocol population. We analyzed safety data in the safety population.

4.1 Inclusion Criteria

- Aged 18 to 75 years
- Karnofsky Performance Status (KPS) ≥ 70
- Hepatocellular carcinoma diagnosed pathologically or clinically based on the American Association for the Study of Liver Diseases practice guideline[35]

- Classified as Barcelona Clinic Liver Cancer (BCLC) Stage C
- Without any previous treatment
- At least 1 measurable intrahepatic lesion according to Response Evaluation Criteria in Solid Tumors version 1.1
- Absence of cirrhosis, or cirrhosis classified as Child-Pugh class A, without ascites and without severe liver atrophy
- No hepatoprotective or supportive therapy within 2 weeks prior to enrollment, and meeting the following criteria: Granulocyte count ≥ 1,500/μL, Platelet count ≥ 75,000/μL, Hemoglobin ≥ 8.5g/dL, Total bilirubin < 30mmol/L, Serum albumin > 3.2 g/dL, ASL and AST ≤ 5 × upper limit of normal, Serum creatinine ≤1.5 × upper of normal, 0.8 < INR < 1.2 or prothrombin time prolongation ≤ 6 seconds.
- Signed informed consent document

4.2 Exclusion Criteria

- History of concurrent cardiac disease, gastrointestinal bleeding within 1-month, severe infection (> grade 2 National Cancer Institute [NCI]-Common Terminology Criteria for Adverse Events [CTCAE] version 5.0), or other severe comorbidities that preclude tolerance to treatment
- Known history of other malignant tumors
- Prior treatment with anti-PD-1 monoclonal antibody
- Known history of hypersensitivity to relevant drugs
- Known history of organ allograft
- Pregnant or lactating women
- Other factors that may affect patient enrollment and outcome evaluation
- Patients assessed by the investigator as having poor compliance, unable to ensure adherence to the study protocol regarding medication and follow-up

5. Treatment and Administration

Patients received sorafenib (400 mg orally twice daily) continuously. Toripalimab (240 mg by intravenous infusion) was administered every three weeks.

HAIC was performed once every three weeks for a total of six cycles. The procedures of HAIC were shown as follows. An arterial catheter was inserted using an image-guided procedure through the femoral artery. The femoral artery was percutaneously punctured using the Seldinger's technique. A 5 French catheter will be inserted into the celiac trunk or superior mesenteric artery for arteriography. Then, a 2.7 French microcatheter will be superselectively placed into the feeding arteries of the tumor and the tumor thrombus. If the tumors simultaneously accept blood supply from the celiac trunk and superior mesenteric artery, the microcatheter will be placed into the largest tumor feeding arteries. When blood flow into the gastroduodenal artery was confirmed by micro-catheter angiography, the route was embolized with a coil or micro-coil to prevent reflux of chemotherapeutic drugs to the stomach and duodenum. The peripheral end of the micro-catheter will be locked with a heparin lock (10 ml, 10,000 units, 1: 1,000 dilution) to prevent clotting of the catheter. The peripheral part of the catheter exposed outside the body will be covered with medical sterile gauze and fastened on the skin of the thigh using medical rubberized fabric and a bandage. Then, the patient will be transferred to the ward and confined to bed for over 24 hours. After confirming the location of the tips of the microcatheter by bed side X-ray radiography, the microcatheter was marked in vitro and connected to the artery infusion pump to administer the chemotherapy agent. When the mark changed, bedside X-ray radiography was also conducted to confirm the location of the catheter tip. If dislocation of the catheter tip was confirmed, the patient was transferred to the digital subtraction angiography room to correct the location of the catheter tip. After HAIC is completed, the catheter and sheath will be removed. No implanted port catheter system was used and repetitive femoral artery puncture and catheterization was performed in the next HAIC cycle. After transferring to the inpatient ward, participants received drug infusion

via the hepatic artery as follows: oxaliplatin, 85 mg/m² from hour 0 to 2 on day 1; leucovorin, 400 mg/m² from hour 2 to 3 on day 1; and fluorouracil, 400 mg/m² bolus at hour 3 and 2400 mg/m² over 24 hours.

In this study, in addition to the trial interventions, the following management principles were applied unless otherwise specified: 1) Routine testing for hepatitis B virus DNA load was performed before treatment. Active antiviral therapy was recommended for all enrolled patients. 2) All forms of immunotherapy other than the study treatment including adoptive immunocyte infusion therapies such as CIK, and immune cytokine therapies such as thymosin were restricted during the study period. 3) During the study treatment period, concurrent surgical resection, systemic chemotherapy, and radiotherapy were not permitted, although traditional Chinese herbal preparations and other nutritional supportive therapies were allowed.

Study treatment was terminated for any of the following reasons: radiological disease progression confirmed at two consecutive visits within three months of treatment; occurrence of severe adverse reactions requiring treatment discontinuation due to intolerability; tumor shrinkage on imaging to an extent allowing surgical resection; deterioration of general condition or liver function meeting exclusion criteria; onset of neurological symptoms leading to functional impairment, or other life-threatening adverse reactions.

After meeting the criteria for study discontinuation, subsequent treatment was chosen by the attending physician based on the patient's condition, without restrictions. Patients with evident disease progression were advised to receive supportive care. For those eligible for surgical resection, tumor resection was performed, followed by regular surveillance; in case of recurrence, appropriate treatments—including surgery, radiofrequency ablation, intervention, or supportive care—were administered. In cases of severe adverse reactions, HAIC, sorafenib, or toripalimab was discontinued, and symptomatic management was provided.

6. Dose Modification

Dose reduction, criteria for dose reduction, and criteria for treatment delay in case of adverse reactions to sorafenib were implemented in accordance with the sorafenib prescribing information and referenced literature[3].

6.1 Treatment Interruptions for toripalimab

Administration of toripalimab should be withheld until resolution under the following conditions: 1) Occurrence of severe/life-threatening adverse reactions. 2) If unexplained respiratory symptoms such as non-productive cough, dyspnea, bibasilar crackles, or pulmonary infiltrates radiologically identified develop, toripalimab should be discontinued until further pulmonary evaluation excludes the possibility of interstitial lung disease. 3) If abnormal liver function tests or portal hypertension cannot be clearly attributed to underlying cirrhosis or tumor progression, the possibility of immune-mediated hepatitis induced by toripalimab should be considered.

6.2 Treatment Interruptions for HAIC

HAIC should be delayed until resolution under the following conditions: 1) Severe/life-threatening (grade 4) diarrhea or bloody diarrhea. 2) Severe (grade 3-4) neutropenia with neutrophil count <1000/μL, severe (grade 3-4) thrombocytopenia with platelet count <50,000/μL, and patients with rapidly declining blood counts. 3) Neurological symptoms (sensory impairment, convulsions) persisting for more than 7 days and of significant severity. 4) Signs and symptoms of reversible posterior leukoencephalopathy syndrome (RPLS, also known as posterior reversible encephalopathy syndrome, PRES), which may include headache, altered mental status, seizures, visual disturbances (blurring to blindness), with or without hypertension. 5) If unexplained respiratory symptoms such as non-productive cough, dyspnea, bibasilar crackles, or pulmonary infiltrates radiologically identified develop, HAIC should be discontinued until further pulmonary evaluation excludes the possibility of interstitial

lung disease. 6) If abnormal liver function tests or portal hypertension cannot be clearly attributed to liver metastases, the possibility of rare hepatic vascular abnormalities induced by oxaliplatin should be considered. 7) Patients with severe mucosal reactions or prominent pigmentation.

7. Study Procedures

7.1 Before treatment

The following parameters must be assessed prior to treatment:

1) Hematology parameters including hemoglobin, white blood cell count, neutrophil count, red blood cell count, platelet count;

2) 31-item biochemistry panel including AST, ALT, total bilirubin, direct bilirubin, ALP, albumin, creatinine, γ-GTP, Na, K, Cl, amylase, lipase, blood glucose;

3) Tumor markers;

4) Coagulation profile.

These laboratory tests must be completed within 1 week before treatment initiation. Imaging studies (contrast-enhanced CT/MRI) must be performed within 2 weeks before treatment initiation.

7.2 Within Treatment

After initiation of the study treatment, the following laboratory tests should be performed every 3 weeks (±5 days):

1) Hematology parameters including hemoglobin, white blood cell count, neutrophil count, red blood cell count, platelet count;

2) 31-item biochemistry panel including AST, ALT, total bilirubin, direct bilirubin, ALP, albumin, creatinine, γ-GTP, Na, K, Cl, amylase, lipase, blood glucose;

3) Tumor markers;

4) Coagulation profile.

Contrast-enhanced CT/MRI of the liver and non-contrast plus contrast-enhanced CT of the chest should be repeated every 6 weeks after treatment initiation.

7.3 Follow-up

A combination of active and passive follow-up methods will be employed. Patients are required to return to the hospital for regular follow-up assessments according to the study schedule. If a patient fails to return within the specified timeframe, telephone or letter follow-up will be conducted. After discontinuation of the study treatment, patients will be followed up every 3 months for survival status and subsequent treatments until death or the end of the study.

8. Efficacy

8.1 Evaluations

Measurable disease and the response criteria used in this protocol were defined in the RECIST criteria version 1.1. Tumor response was based on radiologic assessment only.

8.2 Endpoints

Primary Endpoint

Progression-free survival rate at 6 months, which was defined as the proportion of patients alive, assessable, and free from progression according to RECIST v1.1at 6 months.

Secondary Endpoints

Overall survival, defined as the time from the treatment initiation to death from any cause.

Progression-free survival, time from treatment initiation to disease progression according to RECIST v1.1 or death from any cause.

Objective response rate, the proportion of patients with complete response or partial

response according to RECIST v1.1.

Duration of response, defined as the time between the date of first confirmed documented response of CR or PR and the date of the first documented tumour progression or death due to any cause, whichever occurred first.

Adverse events were assessed according to the National Cancer Institute Common Terminology Criteria for Adverse Events version 5.0.

9. Safety Evaluations

9.1 Adverse Event

An adverse event is any adverse medical occurrence in a patient or study subject following administration of a medicinal product, but does not necessarily have a causal relationship with the treatment. It may refer to any unfavorable medical event that occurs during the study period, regardless of its relationship to the investigational product, irrespective of severity. The recurrence or worsening of pre-existing medical events is also included.

9.2 Serious Adverse Event

A Serious Adverse Event (SAE) is defined as any of the following occurrences unrelated to disease progression, regardless of treatment attribution:

- Results in death
- Is life-threatening
- Requires inpatient hospitalization or prolongation of existing hospitalization
- Causes persistent or significant disability/incapacity
- Leads to congenital anomalies in the subject’s offspring
- Other medically significant events deemed serious by the investigator based on medical judgment (e.g., exacerbation of comorbidities such as diabetes or hypertension)

Exclusions:

• Hospitalization for non-medical reasons (e.g., insurance reimbursement, social welfare, convalescent care)
• Hospitalization due to chronic conditions unrelated to the study (e.g., diabetes, hypertension)

9.3 Attribution Definitions

An adverse event is considered associated with the use of the drug if the attribution is possible, probable, or very likely by the definitions.

• Doubtful

An adverse event for which an alternative explanation is more likely, e.g., concomitant drug(s), concomitant disease(s), or the relationship in time suggests that a causal relationship is unlikely.

• Possible

An adverse event that might be due to the use of the drug. An alternative explanation, e.g., concomitant drug(s), concomitant disease(s), is inconclusive. The relationship in time is reasonable; therefore, the causal relationship cannot be excluded.

• Probable

An adverse event that might be due to the use of the drug. The relationship in time is suggestive (e.g., confirmed by dechallenge). An alternative explanation is less likely, e.g., concomitant drug(s), concomitant disease(s).

• Very likely

An adverse event that is listed as a possible adverse reaction and cannot be reasonably explained by an alternative explanation, e.g., concomitant drug(s), concomitant disease(s). The relationship in time is very suggestive.

9.4. Intensity of AE

All AEs was graded according to the Common Terminology Criteria of Adverse Event (CTCAE), version 4.03 grading scale.

Grade refers to the severity of the AE.

Grade		Description
1	Mild	Symptoms causing no or minimal inference with usual social & functional activities
2	Moderate	Symptoms causing greater than minimal interference with usual social & functional activities
3	Severe	Symptoms causing inability to perform usual social & functional activities
4	Life-threatening	Symptoms causing inability to perform basic self-care functions or medical or operative intervention indicated to prevent permanent impairment, persistent disability
5	Death	Death

10. Data Management

10.1 Data Collection

Clinical and radiological data were prospectively collected from the medical record and our database. The data including, but not limited to, the following would be recorded and analyzed: gender, age, ECOG PS score, positive or negative of hepatitis B surface antigen, alpha-fetoprotein (AFP) level, des-gamma-carboxy prothrombin, albumin-bilirubin (ALBI) grade, alanine aminotransferase, aspartate aminotransferase, albumin, total bilirubin, tumor size, tumor number, absence or presence of portal vein tumor thrombus (PVTT), absence or presence of hepatic vein tumor thrombus (HVTT), absence or presence of extrahepatic metastasis.

10.2 Statistical Analysis

The primary statistical analysis was performed based on the intention-to-treat (ITT) principle using the ITT population, with additional stratified analysis conducted on the data. Data were described without intergroup comparisons. Continuous variables

following a normal distribution were presented as mean ± S.D., while those not normally distributed were expressed as median (range). Overall survival and disease-free survival rates were calculated using the Kaplan–Meier method, without between-group comparisons. All research data were entered into computers and analyzed with SPSS and R studio.

10.3 Tumor Response

Tumor assessments were evaluatd by two investigators based on RECIST v1.1 and mRECIST, respectively. In case of a discrepancy between the two investigators, the tumor response would be assessed by a third, more experienced radiologist who was blinded to the participants treatment information.

11. Ethical Considerations

11.1 Protection of Patients' Rights

This clinical study was conducted in strict accordance with the ethical principles set forth in the Declaration of Helsinki (2000 version) and complied with relevant Chinese laws and regulations. The study was initiated only after the study protocol had been approved by the Ethics Committee of the lead institution responsible for this clinical trial.

11.2 Informed Consent

Prior to enrollment, investigators should inform the patient (or their legal guardian) of the trial procedures, benefits, risks, and patient rights, and must clearly convey that the patient has the right to withdraw from the study at any time without providing any reason. After being fully informed and providing consent, the patient shall sign the Informed Consent Form for confirmation. The informed consent form is prepared in

two copies, to be retained by the subject and the investigator respectively.

11.3 Protection of Personal Information and Identification of Patients

To protect the privacy of individual patients, numbers issued on patients was used to identify or refer to patients. All researchers made the utmost effort to protect personal information.

11.4 Conflicts of Interest

The researchers declare that they have no conflict of interest. The study has no commercial affiliations with any company.

11.5 Institutional Review Board (IRB)

The investigator provided the IRB with current and complete copies of the documents, which include, but are not limited to, final protocol, informed consent, investigators' curriculum vitae, information regarding funding, and other potential conflicts of interest.

12. Appendices

12.1 Tumor Assessment

Overall response, including assessment of the change in tumor burden inside and outside the liver, were assessed by investigators by using the Response Evaluation Criteria in Solid Tumors (RECIST) version 1.1[37]. Assessments were made based on changes in the diameter of tumors that are observed by contrast CT or MRI until completion or discontinuation of the protocol treatment. The objective response rate (ORR) is defined as the rate of CR plus PR. Tumor response includes assessment of target lesions, nontarget lesions and new lesions. All objective responses were

confirmed at least 4 weeks after the first observation.

In addition, the overall response was also assessed according to the modified RECIST (mRECIST) guidelines[38]. Assessments were made based on changes in the diameter of surviving tumors deemed viable by contrast CT or MRI.

Table 12-1. Assessment of Target Lesion Response: Conventional RECIST and mRECIST Assessment for HCC Following the AASLD-JNCI Guideline

RECIST	mRECIST
CR=Disappearance of all target lesions	CR=Disappearance of any intratumoral arterial enhancement in all target lesions
PR=At least a 30% decrease in the sum of diameters of target lesions, taking as reference the baseline sum of the diameters of target lesions	PR=At least a 30% decrease in the sum of diameters of viable (enhancement in the arterial phase) target lesions, taking as reference the baseline sum of the diameters of target lesions
SD=Any cases that do not qualify for either partial response or progressive disease	SD=Any cases that do not qualify for either partial response or progressive disease
PD=An increase of at least 20% in the sum of the diameters of target lesions, taking as reference the smallest sum of the diameters of target lesions recorded since treatment started	PD=An increase of at least 20% in the sum of the diameters of viable (enhancing) target lesions, taking as reference the smallest sum of the diameters of viable (enhancing) target lesions recorded since treatment started

AASLD, American Association for the Study of Liver Diseases; JNCI, Journal of the National Cancer Institute; HCC, hepatocellular carcinoma; mRECIST, modified Response Evaluation Criteria in Solid Tumors; CR, complete response; PR, partial response; SD, stable disease; PD, progressive disease.

Table 12-2. Overall Response Assessment in mRECIST: Responses for All Possible

Combinations of Tumor Responses in Target and Nontarget Lesions with or without the Appearance of New Lesions

Target Lesions	Nontarget Lesions	New Lesions	Overall Response
CR	CR	No	CR
CR	IR/SD	No	PR
PR	Non-PD	No	PR
SD	Non-PD	No	SD
PD	Any	Yes or no	PD
Any	PD	Yes or no	PD
Any	Any	Yes	PD

mRECIST, modified Response Evaluation Criteria in Solid Tumors; CR, complete response; PR, partial response; IR, incomplete response; SD, stable disease; PD, progressive disease.

12.2 Definitions of Eastern Cooperative Oncology Group Performance Status

Table 12-3

Grade	Performance Status
0	Fully active, able to carry on all predisease performance without restriction
1	Restricted in physically strenuous activity but ambulatory and able to carry out work of a light or sedentary nature, e.g., light house work, office work
2	Ambulatory and capable of all selfcare but unable to carry out any work activities; up and about more than 50% of waking hours
3	Capable of only limited selfcare; confined to bed or chair more than 50% of waking hours
4	Completely disabled; cannot carry on any selfcare; totally confined to bed or chair
5	Dead

12.3 Child–Pugh Score[*]

Copyright 1973, copyright British Journal of Surgery Society Ltd. Reproduced with permission. Permission is granted by John Wiley & Sons Ltd on behalf of BJSS Ltd.

Table 12-4

Measure	1 point	2 points	3 points
**Total bilirubin, µmol/L (mg/dL)**	<34 (<2)	34–50 (2–3)	>50 (>3)
**Serum albumin, g/dL**	>3.5	2.8–3.5	<2.8
**Prothrombin time, prolongation (s) or INR**	<4.0<br><1.7	4.0–6.0<br>1.7-2.3	> 6.0<br>>2.3
**Ascites**	None	Mild (or suppressed with medication)	Moderate to severe (or refractory)
**Hepatic encephalopathy[†]**	None	Grade I–II	Grade III–IV

* Child–Pugh A: 5 or 6 points; Child–Pugh B: 7–9 points; Child–Pugh C: >9 points

Grade of encephalopathy:

Grade 0: Lucid, normal personality, normal neurological test results, normal electroencephalogram

Grade 1: Restlessness, sleep disorder, irritability/agitation, tremors, dysgraphia, 5 cps waves

Grade 2: Lethargy, disorientation (temporal), inappropriateness, difficulty maintaining stable posture, ataxia, slow triphasic waves

Grade 3: Somnolence, confused state, disorientation (spatial), hyperreflexia, rigidity, slow waves Grade 4: Coma, no personality/unresponsive, cessation of cerebral activity, slow 2–3 cps delta activity

12.4 BCLC Staging System

Copyright 2007, reproduced with permission from Elsevier

Table 12-5

	**Very early stage (0)**	**Early stage (A)**	**Intermediate stage (B)**	**Advanced stage (C)**	**Terminal stage (D)**
Child–Pugh	A	A-B	A-B	A-B	C
Performance status	0	0	0	1-2	>2
Tumor Features	1 HCC <2cm Carcinoma in situ	1 HCC or 3 Nodules <3cm	Multinodular	Portal invasion, N1, M1	Any

N1, lymph node metastasis. M1, extrahepatic spread.

13. Reference

[1]. Park JW, Chen M, Colombo M, et al. Global patterns of hepatocellular carcinoma management from diagnosis to death: the BRIDGE Study. Liver Int 2015; 35(9): 2155-66.
[2]. Cabibbo G, Enea M, Attanasio M, Bruix J, Craxi A, Camma C. A meta-analysis of survival rates of untreated patients in randomized clinical trials of hepatocellular carcinoma. Hepatology (Baltimore, Md) 2010; 51(4): 1274-83.
[3]. Llovet JM, Ricci S, Mazzaferro V, et al. Sorafenib in advanced hepatocellular carcinoma. The New England journal of medicine 2008; 359(4): 378-90.
[4]. Cheng AL, Kang YK, Chen Z, et al. Efficacy and safety of sorafenib in patients in the Asia-Pacific region with advanced hepatocellular carcinoma: a phase III randomised, double-blind, placebo-controlled trial. Lancet Oncol 2009; 10(1): 25-34.
[5]. Bruix J, Sherman M, American Association for the Study of Liver D. Management of hepatocellular carcinoma: an update. Hepatology (Baltimore, Md) 2011; 53(3): 1020-2.
[6]. European Association For The Study Of The L, European Organisation For R, Treatment Of C. EASL-EORTC clinical practice guidelines: management of hepatocellular carcinoma. J Hepatol 2012; 56(4): 908-43.
[7]. Kudo M, Matsui O, Izumi N, et al. JSH Consensus-Based Clinical Practice Guidelines for the Management of Hepatocellular Carcinoma: 2014 Update by the Liver Cancer Study Group of Japan. Liver Cancer 2014; 3(3-4): 458-68.
[8]. Bruix J, Cheng AL, Meinhardt G, Nakajima K, De Sanctis Y, Llovet J. Prognostic factors and predictors of sorafenib benefit in patients with hepatocellular carcinoma: Analysis of two phase III studies. J Hepatol 2017; 67(5): 999-1008.
[9]. Alkhatib A, Gomaa A, Allam N, Rewisha E, Waked I. Real Life Treatment of Hepatocellular Carcinoma: Impact of Deviation from Guidelines for Recommended Therapy. Asian Pacific journal of cancer prevention : APJCP 2015; 16(16): 6929-34.
[10]. Akiyama M, Miyaaki H, Miuma S, et al. Significance of trans-hepatic arterial chemotherapy for advanced hepatocellular carcinoma with portal vein tumor thrombus. Oncol Rep 2008; 20(2): 353-7.
[11]. Luo J, Guo RP, Lai EC, et al. Transarterial chemoembolization for unresectable hepatocellular carcinoma with portal vein tumor thrombosis: a prospective comparative study. Annals of surgical oncology 2011; 18(2): 413-20.
[12]. Chern MC, Chuang VP, Liang CT, Lin ZH, Kuo TM. Transcatheter arterial chemoembolization for advanced hepatocellular carcinoma with portal vein invasion: safety, efficacy, and prognostic factors. Journal of vascular and interventional radiology : JVIR 2014; 25(1): 32-40.
[13]. Lee HS, Kim JS, Choi IJ, Chung JW, Park JH, Kim CY. The safety and efficacy of transcatheter arterial chemoembolization in the treatment of patients with hepatocellular carcinoma and main portal vein obstruction. A prospective controlled study. Cancer 1997; 79(11): 2087-94.
[14]. Shi M, Lu LG, Fang WQ, et al. Roles played by chemolipiodolization and embolization in chemoembolization for hepatocellular carcinoma: single-blind, randomized trial. Journal of

the National Cancer Institute 2013; 105(1): 59-68.
[15]. Kim HY, Kim JD, Bae SH, et al. A comparative study of high-dose hepatic arterial infusion chemotherapy and transarterial chemoembolization using doxorubicin for intractable, advanced hepatocellular carcinoma. Korean J Hepatol 2010; 16(4): 355-61.
[16]. Sumie S, Yamashita F, Ando E, et al. Interventional radiology for advanced hepatocellular carcinoma: comparison of hepatic artery infusion chemotherapy and transcatheter arterial lipiodol chemoembolization. AJR American journal of roentgenology 2003; 181(5): 1327-34.
[17]. Tsai WL, Lai KH, Liang HL, et al. Hepatic arterial infusion chemotherapy for patients with huge unresectable hepatocellular carcinoma. PloS one 2014; 9(5): e92784.
[18]. Lin CP, Yu HC, Cheng JS, et al. Clinical effects of intra-arterial infusion chemotherapy with cisplatin, mitomycin C, leucovorin and 5-flourouracil for unresectable advanced hepatocellular carcinoma. J Chin Med Assoc 2004; 67(12): 602-10.
[19]. Qin S, Bai Y, Lim HY, et al. Randomized, multicenter, open-label study of oxaliplatin plus fluorouracil/leucovorin versus doxorubicin as palliative chemotherapy in patients with advanced hepatocellular carcinoma from Asia. Journal of clinical oncology : official journal of the American Society of Clinical Oncology 2013; 31(28): 3501-8.
[20]. He MK, Zou RH, Li QJ, et al. Phase II Study of Sorafenib Combined with Concurrent Hepatic Arterial Infusion of Oxaliplatin, 5-Fluorouracil and Leucovorin for Unresectable Hepatocellular Carcinoma with Major Portal Vein Thrombosis. Cardiovasc Intervent Radiol 2018.
[21]. Lyu N, Kong Y, Mu L, et al. Hepatic arterial infusion of oxaliplatin plus fluorouracil/leucovorin vs. sorafenib for advanced hepatocellular carcinoma. J Hepatol 2018; 69(1): 60-9.
[22]. Zhu AX, Finn RS, Edeline J, et al. Pembrolizumab in patients with advanced hepatocellular carcinoma previously treated with sorafenib (KEYNOTE-224): a non-randomised, open-label phase 2 trial. Lancet Oncol 2018; 19(7): 940-52.
[23]. Weber JS, D'Angelo SP, Minor D, et al. Nivolumab versus chemotherapy in patients with advanced melanoma who progressed after anti-CTLA-4 treatment (CheckMate 037): a randomised, controlled, open-label, phase 3 trial. Lancet Oncol 2015; 16(4): 375-84.
[24]. Motzer RJ, Escudier B, McDermott DF, et al. Nivolumab versus Everolimus in Advanced Renal-Cell Carcinoma. N Engl J Med 2015; 373(19): 1803-13.
[25]. Whiteside TL, Demaria S, Rodriguez-Ruiz ME, Zarour HM, Melero I. Emerging Opportunities and Challenges in Cancer Immunotherapy. Clin Cancer Res 2016; 22(8): 1845-55.
[26]. Topalian SL, Drake CG, Pardoll DM. Immune checkpoint blockade: a common denominator approach to cancer therapy. Cancer Cell 2015; 27(4): 450-61.
[27]. Xu C, Chen YP, Du XJ, et al. Comparative safety of immune checkpoint inhibitors in cancer: systematic review and network meta-analysis. BMJ 2018; 363: k4226.
[28]. Chen R, Tao Y, Xu X, et al. The efficacy and safety of nivolumab, pembrolizumab, and atezolizumab in treatment of advanced non-small cell lung cancer. Discov Med 2018; 26(143): 155-66.
[29]. Ayers M, Lunceford J, Nebozhyn M, et al. IFN-gamma-related mRNA profile predicts clinical response to PD-1 blockade. J Clin Invest 2017; 127(8): 2930-40.

[30]. Sharma P, Hu-Lieskovan S, Wargo JA, Ribas A. Primary, Adaptive, and Acquired Resistance to Cancer Immunotherapy. Cell 2017; 168(4): 707-23.
[31]. Xu X, Huang Z, Zheng L, Fan Y. The efficacy and safety of anti-PD-1/PD-L1 antibodies combined with chemotherapy or CTLA4 antibody as a first-line treatment for advanced lung cancer. Int J Cancer 2018; 142(11): 2344-54.
[32]. Menard C, Martin F, Apetoh L, Bouyer F, Ghiringhelli F. Cancer chemotherapy: not only a direct cytotoxic effect, but also an adjuvant for antitumor immunity. Cancer Immunol Immunother 2008; 57(11): 1579-87.
[33]. Kim YH, Choi BK, Kim KH, Kang SW, Kwon BS. Combination therapy with cisplatin and anti-4-1BB: synergistic anticancer effects and amelioration of cisplatin-induced nephrotoxicity. Cancer Res 2008; 68(18): 7264-9.
[34]. Galluzzi L, Buque A, Kepp O, Zitvogel L, Kroemer G. Immunological Effects of Conventional Chemotherapy and Targeted Anticancer Agents. Cancer Cell 2015; 28(6): 690-714.
[35]. Bruix J, Sherman M, Practice Guidelines Committee AAftSoLD. Management of hepatocellular carcinoma. Hepatology 2005; 42(5): 1208-36.
[36]. He M, Li Q, Zou R, et al. Sorafenib Plus Hepatic Arterial Infusion of Oxaliplatin, Fluorouracil, and Leucovorin vs Sorafenib Alone for Hepatocellular Carcinoma With Portal Vein Invasion: A Randomized Clinical Trial. JAMA oncology 2019.
[37]. Tsuchida Y, Therasse P: Response evaluation criteria in solid tumors (RECIST): new guidelines. Med Pediatr Oncol 37:1-3, 2001.
[38]. Lencioni R, Llovet JM: Modified RECIST (mRECIST) assessment for hepatocellular carcinoma. Semin Liver Dis 30:52-60, 2010.

STROBE Statement—checklist of items that should be included in reports of observational studies

	Item No	Recommendation	
**Title and abstract**	1	(*a*) Indicate the study's design with a commonly used term in the title or the abstract	1
		(*b*) Provide in the abstract an informative and balanced summary of what was done and what was found	3
**Introduction**			
Background/rationale	2	Explain the scientific background and rationale for the investigation being reported	4
Objectives	3	State specific objectives, including any prespecified hypotheses	5
**Methods**			
Study design	4	Present key elements of study design early in the paper	15
Setting	5	Describe the setting, locations, and relevant dates, including periods of recruitment, exposure, follow-up, and data collection	5
Participants	6	(*a*) *Cohort study*—Give the eligibility criteria, and the sources and methods of selection of participants. Describe methods of follow-up<br>*Case-control study*—Give the eligibility criteria, and the sources and methods of case ascertainment and control selection. Give the rationale for the choice of cases and controls<br>*Cross-sectional study*—Give the eligibility criteria, and the sources and methods of selection of participants	15
		(*b*) *Cohort study*—For matched studies, give matching criteria and number of exposed and unexposed<br>*Case-control study*—For matched studies, give matching criteria and the number of controls per case	
Variables	7	Clearly define all outcomes, exposures, predictors, potential confounders, and effect modifiers. Give diagnostic criteria, if applicable	16
Data sources/ measurement	8*	For each variable of interest, give sources of data and details of methods of assessment (measurement). Describe comparability of assessment methods if there is more than one group	18
Bias	9	Describe any efforts to address potential sources of bias	15
Study size	10	Explain how the study size was arrived at	17
Quantitative variables	11	Explain how quantitative variables were handled in the analyses. If applicable, describe which groupings were chosen and why	
Statistical methods	12	(*a*) Describe all statistical methods, including those used to control for confounding	17
		(*b*) Describe any methods used to examine subgroups and interactions	
		(*c*) Explain how missing data were addressed	
		(*d*) *Cohort study*—If applicable, explain how loss to follow-up was addressed<br>*Case-control study*—If applicable, explain how matching of cases and controls was addressed<br>*Cross-sectional study*—If applicable, describe analytical methods taking account of sampling strategy	
		(*e*) Describe any sensitivity analyses	

Continued on next page

**Results**			
Participants	13*	(a) Report numbers of individuals at each stage of study—eg numbers potentially eligible, examined for eligibility, confirmed eligible, included in the study, completing follow-up, and analysed	5, 7
		(b) Give reasons for non-participation at each stage	5
		(c) Consider use of a flow diagram	5
Descriptive data	14*	(a) Give characteristics of study participants (eg demographic, clinical, social) and information on exposures and potential confounders	5
		(b) Indicate number of participants with missing data for each variable of interest	
		(c) *Cohort study*—Summarise follow-up time (eg, average and total amount)	5
Outcome data	15*	*Cohort study*—Report numbers of outcome events or summary measures over time	7, 8
		Case-control study—Report numbers in each exposure category, or summary measures of exposure	
		Cross-sectional study—Report numbers of outcome events or summary measures	
Main results	16	(*a*) Give unadjusted estimates and, if applicable, confounder-adjusted estimates and their precision (eg, 95% confidence interval). Make clear which confounders were adjusted for and why they were included	5-11
		(*b*) Report category boundaries when continuous variables were categorized	
		(*c*) If relevant, consider translating estimates of relative risk into absolute risk for a meaningful time period	
Other analyses	17	Report other analyses done—eg analyses of subgroups and interactions, and sensitivity analyses	
**Discussion**			
Key results	18	Summarise key results with reference to study objectives	11
Limitations	19	Discuss limitations of the study, taking into account sources of potential bias or imprecision. Discuss both direction and magnitude of any potential bias	13
Interpretation	20	Give a cautious overall interpretation of results considering objectives, limitations, multiplicity of analyses, results from similar studies, and other relevant evidence	11-13
Generalisability	21	Discuss the generalisability (external validity) of the study results	14
**Other information**			
Funding	22	Give the source of funding and the role of the funders for the present study and, if applicable, for the original study on which the present article is based	19

*Give information separately for cases and controls in case-control studies and, if applicable, for exposed and unexposed groups in cohort and cross-sectional studies.

Note: An Explanation and Elaboration article discusses each checklist item and gives methodological background and published examples of transparent reporting. The STROBE checklist is best used in conjunction with this article (freely available on the Web sites of PLoS Medicine at http://www.plosmedicine.org/, Annals of Internal Medicine at http://www.annals.org/, and Epidemiology at http://www.epidem.com/). Information on the STROBE Initiative is available at www.strobe-statement.org.
